# CoVeriTest with Dynamic Partitioning of the Iteration Time Limit (Competition Contribution)

**DOI:** 10.1007/978-3-030-45234-6_30

**Published:** 2020-03-13

**Authors:** Marie-Christine Jakobs

**Affiliations:** 8 University of Paderborn, Paderborn, Germany; 9 ICREA, Open University of Catalonia, Barcelona, Spain; Department of Computer Science, Technical University of Darmstadt, Darmstadt, Germany

**Keywords:** Test-case generation, Cooperative verification, CPAchecker

## Abstract

Our CoVeriTest submission, which is implemented in the analysis framework CPAchecker, uses verification techniques for automatic test-case generation. To this end, it checks the reachability of every test goal and generates one test case per reachable goal. Instead of checking the reachability of every test goal individually, which is too expensive, CoVeriTest considers all test goals at once and removes already covered goals from future reachability queries. To deal with the diverse set of Test-Comp tasks, CoVeriTest uses a hybrid approach that interleaves value and predicate analysis. In contrast to Test-Comp’19, the time limit per iteration is no longer fixed for an analysis. Instead, we fix the iteration time limit and split it dynamically among the analyses, rewarding analyses that previously covered more test goals per time unit.

## 1 Test-Generation Approach

Test-case generation approaches have different strengths and weaknesses. To deal with the diverse Test-Comp benchmark, we therefore use an hybrid approach. More concrete, our Test-Comp'20 submission CoVeriTest combines different verification approaches using the idea of **co**operative, **veri**fier-based **test**ing [6].

Figure 1 shows the workflow of our CoVeriTest submission. Like in Test-Comp'19, CoVeriTest iteratively combines a value analysis [5], which only tracks the explicit values of those variables stored in its precision, and a predicate analysis, which applies adjustable block encoding [4] and abstracts at loop heads only. Both analyses use counterexample-guided abstraction refinement [8] to adjust their precision (the set of tracked variables or the set of predicates) and check which open test goals can be reached. Whenever one analysis reaches a test goal, i.e., it finds a real counterexample, a test case adhering to the Test-Comp exchange format[1] is constructed from that counterexample [1] and the test goal

[*] This work was funded by the Hessian LOEWE initiative within the Software-Factory 4.0 project.

[**] jury-member

[1] https://gitlab.com/sosy-lab/software/test-format/tree/master

H. Wehrheim and J. Cabot (Eds.): FASE 2020, LNCS 12076, pp. 540–544, 2020.
https://doi.org/10.1007/978-3-030-45234-6_30

is removed from the set of open test goals. Depending on the Test-Comp'20 property, the set of test goals is initialized to the set of all `__VERIFIER_error()` calls or the set of all branches.

Like in Test-Comp'19, both analyses resume their exploration from the previous round and do not exchange any further information. The novelty for Test-Comp'20 is the dynamic adjustment of the analyses' time limits. To better adjust to the program, we redistribute the iteration time limit among the analyses after each iteration round. Initially, we grant the value analysis 20 s and the predicate analysis 80 s. Thereafter, we use the normalized progresses $p_V$ and $p_P$ reported by the analyses to compute the new time limits. The normalized progress is the number of test goals covered by the analysis in the round divided by the total number of test goals. If no analysis made progress ($p_V \leq 0$ and $p_P \leq 0$), we will reuse the time limits from the current round. Otherwise, we adjust the limits according to Eq. 1 ($i \in \{V, P\}$). Each analysis gets at least 10 s to avoid to turn it off. The remaining 80 s of the iteration limit are redistributed according to the relative contribution of each analysis. The relative contribution of an analysis is its progress per time limit related to the sum of the progresses per time limit.

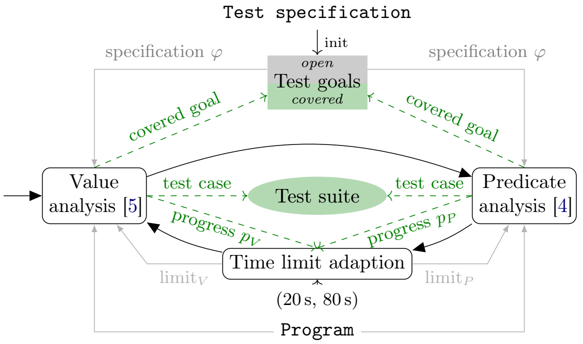

Fig. 1: CoVeriTest workflow for Test-Comp'20

$$\text{limit}_i^{\text{new}} = 10\,\text{s} + \frac{\frac{p_i}{\text{limit}_i}}{\frac{p_V}{\text{limit}_V} + \frac{p_P}{\text{limit}_P}} * 80\,\text{s} \quad (1)$$

The main differences to HybridTiger [11], which also applies cooperative, verifier-based testing, are that HybridTiger uses multi-goal partitioning [10] and that HybridTiger uses fixed time limits 120 s and 720 s for value and predicate analysis.

## 2 Tool Architecture

CoVeriTest is implemented within the Java-based software-analysis framework CPAchecker [3], which uses the Eclipse CDT parser[2] and integrates different SMT solvers via the JavaSMT [9] interface. For Test-Comp'20, we rely on CPAchecker's default SMT solver MathSAT5 [7].

[2] https://www.eclipse.org/cdt/

CPAchecker's core is the configurable program analysis framework [2], which defines the basis for the verification approaches. The framework consists of two parts: configurable program analyses (CPAs) and the CPA algorithm. CPAs like the value and predicate analysis used by CoVeriTest describe program analyses. Therefore, they define the abstract domain and the analysis operators. The CPA algorithm performs the reachability analysis for a given CPA and program.

To integrate further verification techniques, the CPA framework is enhanced with algorithms like counterexample-guided abstraction refinement [8], the circular algorithm, which performs a continuous iteration over a set of analyses, or the test-case generation algorithm. To produce test cases, the test-case generation algorithm wraps and runs another analysis, generates test cases from counterexamples [1] returned by the wrapped analysis, updates the analysis specification (i.e., removes covered goals), and thereafter continues the wrapped analysis.

## 3 Strengths and Weaknesses

CoVeriTest won the third place in the category Cover-Branches and in contrast to Test-Comp'19, became better than KLEE in this category.

The major change of CoVeriTest from Test-Comp'19 to Test-Comp'20 is the dynamic adjustment of the iteration time limits. Thus, many strength and weaknesses are still the same as in Test-Comp'19. CoVeriTest's iterative combination of predicate and value analysis helped to adapt to the diverse set of Test-Comp tasks and its direct search of the test goals lead to few test cases. Also, CoVeriTest has still problems with tasks that contain large arrays because these are not supported by the underlying analyses. Furthermore, CoVeriTest has problems with the new subcategory BusyBox-Memsafety and fails to parse the programs in the new subcategory SQLite-MemSafety.

Now, let us discuss the effect of the adjustment of the time limits. For the time limit adjustment, we use the progress of the analyses measured in number of covered goals. Since there only exists one (reachable) test goal per task in the Cover-Error category, either both analyses make no progress in an iteration ($p_V \leq 0$ and $p_P \leq 0$) or one analysis covered the goal and CoVeriTest stops. Thus, the time limit adjustment has no effect on the Cover-Error category.

Next, let us consider the Cover-Branches category. Our own comparison of the CoVeriTest submissions for Test-Comp'19 and Test-Comp'20 revealed that the time limit adjustment mainly affects tasks of the ECA subcategory. In total, the coverage value for 320 tasks decreased and the coverage value for 591 tasks increased. Moreover, the increase is typically significantly larger than the decrease (on average 6.3 percent points increase compared to 1.5 percent points decrease). Furthermore, most of the tasks with a difference in the coverage value belong to the ECA subcategory. Therefore, the time limit adjustment pays off. Nevertheless, CoVeriTest could still perform better on the ECA subcategory. We believe that one problem in the ECA subcategory are redundant test goals, which lead to the same or similar test case generated multiple times and, thus, a waste of time.

## 4 Setup and Configuration

CoVeriTest is distributed as part of CPAchecker[3], which requires a Java 8 runtime environment. Our Test-Comp'20 submission, with which we participated in all categories, uses CPAchecker in revision `32236`. After the environmental setup, one can run CoVeriTest on program `program.i` with the following command. The file `property.prp` is a placeholder for the test specification, either `coverage-error-call.prp` or `coverage-branches.prp`.

```
scripts/cpa.sh -testcomp20 -benchmark -heap 10000m
    -spec property.prp program.i
```

The command above assumes that `program.i` runs in a 32-bit environment. When requiring a 64-bit environment, one needs to add the parameter `-64` to the above command. Moreover, if the machine has not enough RAM to handle the specified Java heap memory, one can decrease the value passed with `-heap`.

The test suite generated during the execution of CoVeriTest is written to the directory `test-suite`, which is a subdirectory within the output directory of CPAchecker. As defined by the Test-Comp rules, the test suite contains a metadata file and test-case files adhering to the required XML format.

## 5 Project and Contributors

CoVeriTest is a component of the open-source project CPAchecker [3], which is hosted by Dirk Beyer's group at LMU Munich under Apache 2.0. Currently, also members of the Institute for System Programming of the Russian Academy of Sciences, Masaryk University, and Technical University of Darmstadt contribute to CPAchecker. We would like to thank all contributors.
